# Metabolic Conversions by Lactic Acid Bacteria during Plant Protein Fermentations

**DOI:** 10.3390/foods11071005

**Published:** 2022-03-29

**Authors:** Wim Engels, Jamie Siu, Saskia van Schalkwijk, Wilma Wesselink, Simon Jacobs, Herwig Bachmann

**Affiliations:** 1 NIZO, Kernhemseweg 2, 6718 ZB Ede, The Netherlands; jamie.siu@daiyafoods.com (J.S.); saskia.vanschalkwijk@nizo.com (S.v.S.); wilma.wesselink@nizo.com (W.W.); simon.jacobs@nizo.com (S.J.); herwig.bachmann@nizo.com (H.B.); 2 Daiya Foods Inc., 3100 Production Way, Burnaby, BC V5A 4R4, Canada; 3 Systems Biology Lab, Vrije Universiteit, De Boelelaan 1108, 1081 HZ Amsterdam, The Netherlands

**Keywords:** lactic acid bacteria, plant protein, aroma, high throughput screening, enzyme assay, GC-MS, fermentation

## Abstract

To secure a sustainable food supply for the rapidly growing global population, great efforts towards a plant-based diet are underway. However, the use of plant proteins comes with several challenges, such as improvement or removal of undesired flavours, and generation of desired texture properties. Fermentation holds large potential to alter these properties, but compared to dairy fermentations, our knowledge on strain properties in different plant-based substrates is still limited. Here, we explored different lactic acid bacteria for their ability to grow, produce flavour compounds, or remove off-flavour compounds from different plant proteins. For this, 151 LAB strains from dairy and non-dairy origins were cultured in plant protein plus coconut oil emulsions supplemented with glucose. Pea, chickpea, mung, fava, and soybean proteins were used in the study and bacterial strains for screening included the genera *Streptococcus*, *Lactococcus*, *Lactobacillus*, and *Leuconostoc*. Efficient, high throughput, screening on plant proteins was developed and strains were assessed for their ability to (i) acidify and decrease the pH; (ii) express key enzymes involved in the formation of amino acid derived flavours, which included PepN (aminopeptidase N), PepXP (X-prolyl dipeptidyl peptidase), EstA (esterase), BcAT (branched chain aminotransferase), CBL (cystathione beta lyase), and ArAT (aromatic aminotransferase); and (iii) improve the overall aroma profile by generating dairy/cheesy notes and decreasing off flavours. Suitable screening conditions were determined, and highlighted the importance that a sufficient heat treatment must be applied to samples containing plant proteins, prior to fermentation, as an outgrowth of spore forming *Bacillus cereus* was observed if the material was only pasteurised. Enzyme activities for strains measured in rich broth vs. a buffered protein solution showed little-to-no correlation, which illustrated the importance of screening conditions to obtain predictive enzyme measurements. Aroma formation analysis allowed to identify strains that were able to increase key aromas such as diacetyl, acetoin, 2- and 3-methyl butanol, and 2,3-pentanedione, as well as decrease the off-flavours hexanal, pentanal, and nonanal. Our findings illustrate the importance of strain specific differences in the assessed functionalities and how a methodical approach to screening LAB can be applied to select suitable microorganisms that show promise in fermentation of plant proteins when applied in non-dairy cheese applications.

## 1. Introduction

The sustainability of food production has been at the forefront of global discussions in recent years. The global population is estimated to reach 10 billion by 2050 and this population size cannot be sustained with our current dietary habits and its resulting food production methods [1]. The production of animal derived protein comes with many challenges, such as inefficient resource utilisation and waste production, as well as animal welfare concerns. These issues are greatly reduced by moving towards direct use of plant proteins and a diet consisting of more plant-based foods has been touted as a strategy to not only improve human health, but also to improve the overall sustainability of the food supply chain. 

The replacement of animal proteins with plant proteins in food applications has numerous technological hurdles. For example, plant proteins often have lower solubility, contain undesired off aromas, possess poor colour and flavour, and perform with decreased techno-functionality when compared with, for example, conventional milk proteins. As a result, there has been an increase in research to better understand the functionality of plant derived proteins. Such research involves, for instance, assessing and comparing approaches to decrease or remove compounds, such as hexanal and nonanal, that contribute to off notes inherent in these ingredients. Some techniques which have been employed include using direct steam injection, creation of flavour maskers or flavour modulators, and fermentation with lactic acid bacteria (LAB) to reduce off flavours [2].

The process of fermentation has been widely used to preserve food, while also creating distinct aromas and desirable flavours. For common substrates such as milk, the underlying biochemical reactions are well described [3]. During production of yoghurt and cheese, primary microbial metabolism of lactic acid bacteria leads to the production of lactate from lactose, which decreases the pH and, thereby, warrants microbial safety. Through numerous other enzymes (e.g., lipases, aminopeptidases, endopeptidases, and di-tripeptidases) important aromatic and flavour compounds are formed [4]. Interestingly, fermentation has also been employed to remove off-flavour notes from plant proteins [2,5,6].

While, currently, most dairy mimics are not fermented, it is expected that introducing fermentation into the production process would increase the products’ overall consumer acceptability as it may better replicate its dairy counterpart. With the use of plant proteins in place of casein, many challenges arise regarding the formation of cheese-like flavours since the conversion of casein is essential in the development of amino acid derived flavours in cheese. The absence of lactose in the ingredients used to make non-dairy cheeses presents issues since traditional dairy strains are adapted to utilise lactose as a main carbon source for metabolism and growth. The presence of other complex carbohydrates in plant materials, such as pectin, cellulose, and other fibres, may not be easily degraded by enzymes and, thus, their utilisation by LAB may be limited. While plant materials may have these inherent complexities, there are many examples of certain strains of lactic acid bacteria which have successfully been able to grow on plant substrates in other food products. For example, species of the genera *Leuconostoc*, *Weissella*, and *Lactobacillus* have been reported to play a crucial role in the production of vegetable-based products, namely kimchi and sauerkraut [7,8]. *Lactiplantibacillus plantarum* was found to be the predominant LAB species in the creation of the African cassava-based dish, gari [9], and Schindler et al. [2] illustrated that pea protein could successfully be cultured with *L. plantarum* or with *P. pentosaceus* without the use of added carbohydrates, yielding a product with an overall reduction in off notes and improved aroma. Xing et al. [5], explored the potential of using autochthonous LAB found in chickpea flour and chickpea flour derivatives as a means of improving the nutritional properties through solid state fermentation. *P. pentosaceus* and *P. acidilactici* were the predominant strains of LAB found on these materials and through fermentation, they observed a 90% decrease in flatulence-causing α-galactosides, in particular raffinose, stachyose, and verbascose [5].

Although work has been conducted to understand the off flavour removal of plant proteins, there is little research available concerning the potential to create cheese-like aromas through fermentation by LAB. Such knowledge is essential to be able to create, for example, non-dairy cheese analogues [10]. The aim of this research was to screen a broad collection of lactic acid bacteria on various plant substrates (i.e., pea (*Pisum sativum* L.), mung bean (*Vigna radiata*), fava bean (*Vicia faba*), soybean (*Glycine max)*, and chickpea (*Cicer arietinum*) proteins). Fast and reliable monitoring of microbial growth through optical density measurements is not possible in the chosen substrates, therefore, we established an efficient protocol to monitor the culture pH as an indicator for growth over time using a pH sensitive fluorescent probe. For a selection of strains, we determined key flavour enzyme activities in plant protein suspensions including aromatic amino transferase, branched chain amino transferase, PepN, PepXP, cystathionine beta-lyase, and esterase. Finally, GC-MS analysis was applied on emulsions containing plant-protein, coconut oil, and glucose to determine the ability of selected strains to produce desirable volatile compounds and/or to remove off-flavours. 

## 2. Methods & Materials

### 2.1. Ingredients and Raw Materials

Plant proteins used in this study were: pea protein isolate (Pisane C9, Cosucra, Warcoing, Belgium), mung bean protein isolate (GABPRO-M80B+, GAB Foods Co., Hong Kong, China), fava bean protein isolate (GABPRO-F90B, GAB Foods Co., China), soy protein isolate (Soyafarm F, Fuji Oil, Osaka, Japan), and chickpea protein concentrate (Artesa, Nutriati, Henrico, VA, USA). Pure coconut oil (Ertilor 24/26, Fuji Oil Europe, Gent, Belgium) was used to prepare plant protein emulsions. The percentages of protein for the pea, mung, fava, soy, and chickpea protein were 86%, 80%, 88%, 83%, and 59%, respectively, according to the supplier’s certificate of analyses. A 20% glucose stock was purchased from Tritium Microbiologie B.V. (Eindhoven, The Netherlands).

### 2.2. Strain and Inoculum Preparation

A total of 151 bacterial strains isolated mainly from dairy and plant origins were used in this study and obtained from the NIZO culture collection (NIZO Food Research, Ede, The Netherlands). Strains included approximately 45%, 29%, 15%, and 10% of *Lactococcus*, *Lactobacillus*, *Streptococcus*, and *Leuconostoc* species, respectively. For preparing the inoculum, lactococci and streptococci were cultured in M17 broth supplemented with 0.5% glucose (*m*/*v*) (GM-17), and lactobacilli and leuconostocs were cultured in De Man Rogosa and Sharpe (MRS) broth. All media and agar used were obtained from Tritium Microbiologie B.V. (The Netherlands). Strains were incubated in microtiter plates at various conditions: 25 °C, anaerobic, 72 h (*Leuconostoc* strains); 37 °C, 48 h (*Streptococcus* strains); 37 °C, anaerobic, 72 h (thermophilic lactobacilli); 30 °C, 24 h (*Lactococcus* strains); and 30 °C, 48 h (mesophilic lactobacilli). Plate layouts were designed to reduce potential positional effects which could be associated with culturing in 96 well plates and contained duplicates of all strains. 

### 2.3. Plant Emulsion Preparation 

Plant emulsions were made by suspending 5% (*w*/*w*) plant protein in reverse osmosis (RO) water and mixing at room temperature for 15 min. Coconut oil was heated to 30 °C and 5% (*w*/*w*) was added to the plant protein suspension and pre-homogenised using a rotor-stator homogeniser (Polytron PT3100, Kinematica AG, Malters, Switzerland), prior to homogenisation at 30/300 bar (Panda 2k, GEA NIRO Soavi, Parma, Italy). The pH of the emulsions was set to 6.8 using 10% lactic acid prior to autoclaving at 105 °C for 30 min. Plant emulsions for determining acidification were supplemented with 0.5% (*m*/*v*) via a 20% glucose stock and sterile 1% 1.064 mM carboxyfluorescein (Sigma-Aldrich, St. Louis, MO, USA) as described earlier [11]. To minimise the amount of rich medium transferred into the various plant emulsions during inoculation with bacterial strains, the inoculum prepared in rich medium were diluted with plant suspensions. For this, plant protein suspensions per protein type were created by suspending 5% (*w*/*w*) plant protein into RO water and mixing at room temperature for 15 min. The pH of the suspensions was adjusted to 6.8 using 10% lactic acid prior to autoclaving at 105 °C for 30 min. After cooling on ice, the suspensions were supplemented with 0.5% (*m*/*v*) via a 20% glucose stock. 

### 2.4. Screening Strains for Acidification in Plant Emulsions

The various strains were pre-grown in rich media to stationary phase and diluted in 5% plant protein suspensions at 2.5% in 96 well plates. Immediately thereafter, plant protein emulsions were inoculated at 2.5% with these dilutions. To facilitate fast parallel high throughput screening, ninety-six well plates were combined into a 384 well black plates with a transparent bottom (Fluotrac 384, Greiner Bio-One, Kremsmünster, Austria) in duplicate and sealed with an aluminium seal to minimise evaporation. The course of acidification was determined over a 24–48 h period, at appropriate temperatures, by measuring fluorescence kinetically using an incubated spectrophotometer at 520 nm (excitation 485 nm), measuring every 15 min. Acidification measurements were based on the decrease in fluorescent intensity of the pH dependent indicator, 5-(6) carboxyfluorescein. A pH calibration curve was created in each plant protein emulsion with known pH’s by adjusting with 10% lactic acid. Converted values represent an indication of the final pH as the 5-(6) carboxyfluorescein indicator loses sensitivity and signal intensity at more acidic pH values, working best in pH ranges from 5–8 (The Molecular Probes Handbook, ThermoFischer, Waltham, MA, USA).

### 2.5. Enzyme Assays 

Pre-cultures were used to inoculate either fresh broth (GM17 or MRS—referred to as broth samples) or buffered pea suspension (BPS). BPS was prepared by bringing pea protein isolate (1% (*w*/*w*)) in 19 g/L β glycerol phosphate disodium salt pentahydrate (Sigma-Aldrich, USA) solution followed by mixing at room temperature for 15 min. The pH was adjusted to 6.8 using lactic acid prior to autoclaving at 105 °C for 30 min. After cooling, the suspension was supplemented with 0.5% (*m*/*v*) glucose and centrifuged at 10,000 rpm for 15 min. The supernatant was collected and used as the BPS culture medium. Broth samples, 1.5 mL in deep well plates, were inoculated with 1% of pre-culture. To minimise transfer of pre-culture broth to BPS, the pre-culture cell pellets were washed in 1 mL of 0.9% sterile saline solution. The BPS, in deep well plates, was then inoculated with 5% of the washed pre-culture. Broth samples and BPS were incubated at the desired temperature. 

After incubation of broth and pea samples until cells reached stationary phase, cell free extracts were prepared as described earlier [12] with the modifications that bead beating was carried out in 1 mL of buffer with four cycles of bead beating.

Determinations of PepN, PepXP, esterase (EstA), and BcAT activities were carried out by applying specific chromogenic model substrates or by measuring the NADH reduction rate (BcAT), as described earlier [12]. Aromatic amino transferase (ArAT) activities were performed by measuring the formation of indole-3-pyruvate, the keto acid of tryptophan at 327 nm at 37 °C. The assay was carried out as described by [13] with a slight modification using 10 mM of α-ketoglutaric acid potassium in place of 10 mM α-ketoglutarate. The pH of this mixture was adjusted to pH 7.5 and tryptophan was used as the substrate. Cystathionine beta lyase (CBL) activity was quantified as described earlier [14]. Enzyme activities were expressed as nmol substrate converted/min·mL culture as the incubations in the high protein containing suspensions did not allow an accurate estimation of the microbial protein. 

### 2.6. Incubation for Volatile Compound Formation and Analysis Using GC-MS 

Selected strains were grown in plant protein emulsions that consisted of pea, chickpea, or mung bean protein isolates together with coconut oil. The emulsion was prepared using 5% (*w*/*w*) of each plant protein isolate, 5% (*w*/*w*) oil and 0.5% (*m*/*v*) glucose adjusted to pH 6.8 using 10% lactic acid. After mixing, homogenisation (30/300 bar, Panda 2k, GEA Niro Soavi, Italy) and autoclaving (105 °C for 30 min) were performed. Three millilitres of inoculated (with 3% of a pre-culture) emulsion were transferred into sterile 10 mL GC vials, sealed with a screw cap, and incubated for three days at the appropriate temperature, dependent on the strain. Uninoculated emulsions were used as negative controls and were incubated as well. After incubation, samples were stored at −18 °C until GC-MS analyses. 

Volatile flavour compounds produced during fermentation were determined by solid phase microextraction (SPME) gas chromatography mass spectrometry (GC-MS) using a Thermo Scientific TRACE GC Ultra and DSQ-II mass-spectrometer. The solid phase extraction was carried out with a grey SPME fibre (Carboxen/PDMS/Divinylbenzene) for 15 min at 40 °C. Subsequently, the fibre was desorbed for 3 min at 250 °C in splitless mode. The extracted compounds were refocused by cold trapping at −110 °C. Desorption was by heating to 200 °C at 50 °C/s and separation was on a 30 m × 0.25 mm column with a VF-1 ms (film thickness = 1 µm) stationary phase (Agilent Technologies, Santa Clara, CA, USA) from 40 °C, for 2 min, to 160 °C at 10 °C/min and to 250 °C at 50 °C/min. Mass spectra were recorded in full scan over a range of *m*/*z* 30–250.

### 2.7. Microbial Analyses and pH Measurements

To determine the efficacy of the plant protein pasteurisation and autoclaving processes, total colony counts were determined in plant suspensions and emulsions on brain heart infusion agar (BHI) in duplicate after incubation at 37 °C for 24 h. Lactococci and lactobacilli were counted by spot plating dilutions of 10^−2^–10^−5^ on GM-17 and MRS agar, respectively, after two days of incubation at 30 °C [15]. pH values were determined by measuring with a pH electrode, calibrated daily using pH 4 and 7 standards (Biotrode, Metrohm, Switzerland). Strain identification was performed by PCR amplifying 16sRNA using primers V1.1 (5′-GCGGCGTGCCTAATACATGC) and V3.2 (5′-ATCTACGCATTTCACCGCTAC). The amplicons were sequenced using Sanger sequencing at BaseClear Leiden, The Netherlands. Sequences were identified by Blast analysis at https://blast.ncbi.nlm.nih.gov/Blast.cgi (accessed 9 December 2019).

### 2.8. Data Analysis

Kinetic acidification data were analysed in R [16]. For each acidification curve, the Savitzky–Golay filter “sgolayfilt” function from the “signal” package was applied to determine the slope (first derivative) over the complete acidification curve with *n* = 19. The minimum value of this analysis was taken as the maximum rate of acidification. GC data were processed using Xcalibur^TM^ chromatography software (Thermo Fisher Scientific, Waltham, MA, USA) and Microsoft Excel. To visualise the data, heat maps were generated using the R heatmaply package. Enzyme and GC-MS data were log-transformed before visualisation in heatmaps. Within the heatmap function, scaling was applied within substrates (acidification, final pH), enzymes activities, or within volatile compounds (GC-MS data).

## 3. Results

### 3.1. Raw Materials Contain Spore Forming Microorganisms 

In initial experiments, protein suspensions were prepared by pasteurisation at 80 °C for 30 min prior to incubation. This temperature was not exceeded to preserve protein functionality. However, the incubation using these relatively low heat-treated suspensions resulted in microbial growth and we identified colonies from pea protein suspension through 16s RNA sequencing as *Bacillus cereus*. If the same raw material was autoclaved at 105 °C for 30 min, no microbial growth was observed. These results indicate that the raw material contained spore forming microbes that cannot be inactivated by pasteurisation. For this reason, 105 °C, 30 min treatment was applied on the suspensions and emulsions for the remainder of this study.

### 3.2. A pH Sensitive Fluorescent Probe Allows High Throughput Acidification Assay in Plant Protein Suspensions

To allow the high throughput analysis (96 or 384 cultures monitored online in parallel) of microbial acidification in plant protein suspensions, we tested if the application of a pH sensitive fluorescent probe allows to record acidification curves online in a high throughput format. For this we employed 5-(6) carboxyfluorescein, which has been used for the determination of online acidification curves in defined media and milk earlier [11,17]. Our results showed that in pea, chickpea, fava, mung, and soy, we could obtain acidification curves (Supplementary Figure S1) which, when combined with a calibration line made in the same medium, also allowed a conversion to the pH. It is important to note that 5-(6) carboxyfluorescein has the best dynamic range between pH 5–7 and pH values below 5 should therefore be interpreted with care. Overall, the results showed that the fluorescent probe allows for high throughput online monitoring of microbial acidification activity in plant protein suspensions. 

### 3.3. Protein Isolates and Concentrates Contain Low Amounts of Fermentable Carbon

To examine the impact of 0.5% additional glucose on microbial growth in pea suspension a sub-set of 86 strains of *L. lactis* of plant and dairy origin were tested. The results showed that without glucose supplementation, no or hardly any acidification occurred (Supplementary Figure S2). We found similar behaviour on fava bean, mung bean, soy, and chickpea, suggesting that protein concentrates and isolates contain only low amounts of fermentable carbon sources. The results led to the addition of 0.5% glucose to the plant protein suspensions/emulsions in the remainder of the study.

### 3.4. Acidification in Plant Emulsions Shows Big Biodiversity

**Figure 1:**
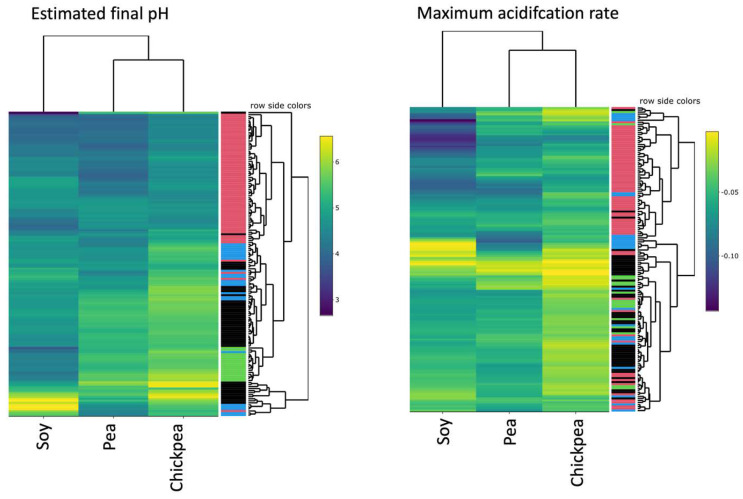
Estimated final pH and the maximum acidification rate determined for 148 bacterial strains (each branch on the right hand dendrogram represents one strain) when grown in soy, pea, or chickpea emulsion supplemented with 0.5% glucose. Values are the mean of four biological replicates. The colours on the right-hand side dendrogram indicate *Lactococcus* (red), *Lactobacillus* (black), *Streptococcus* (blue), and *Leuconostoc* (green). For the estimated final pH, a darker colour indicates a lower pH at the end of the fermentation. For the maximum acidification rate, a darker colour (lower value) indicates a faster acidification rate (a faster drop in pH results in a more negative acidification rate).

Pea, chickpea, and soy emulsions supplemented with 0.5% glucose and 10 mM 5-(6) carboxyfluorescein were inoculated with 148 different lactic acid bacteria and their maximum acidification rates and final pH were determined. The results showed large differences between strains with the final pH ranging from above 6 to below 4 and the acidification rate ranging from below −0.02 pH/h to more than −0.12 pH/h (Figure 1). The strains used for this screening were selected broadly to reflect important species used in food fermentations. The final pH of this screen shows that lactococci tend to reach the lowest final values while streptococci, lactobacilli, and leuconostocs have increasingly higher end pH values. For the acidification rates, *Lactococcus* seems to perform best, particularly in soy. There is no clear trend between *Streptococcus*, *Lactobacillus*, and *Leuconostoc* in this assay. Overall, the data show a broad range of biodiversity within this selected collection of strains. 

### 3.5. Enzyme Activity in Rich Medium Is Not Predictive for Product Environment 

**Figure 2:**
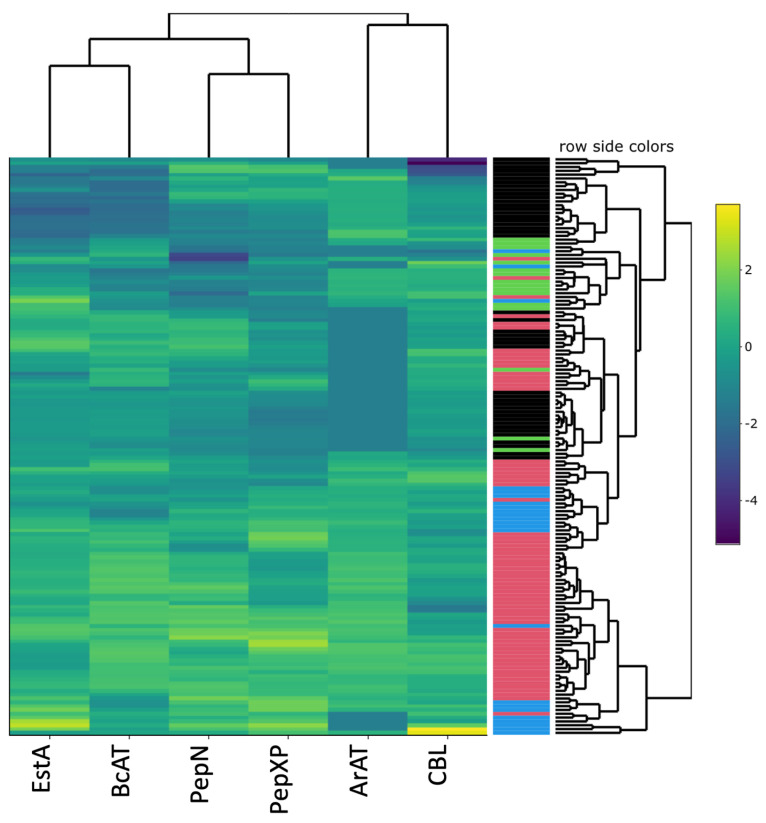
Enzyme activities measured in 151 lactic acid bacteria after growth in a BPS supplemented with 0.5% glucose. Values are the mean of two biological replicates. The row side colours in the heatmap indicate the genus of the bacteria, *Lactococcus* (red), *Lactobacillus* (black), *Streptococcus* (blue), and *Leuconostoc* (green).

To investigate the expression of key enzymes involved in amino acid derived flavour formation, 151 selected strains were grown in two different media, a nutrient rich broth (GM-17 or MRS) and a buffered pea suspension (BPS). PepN, PepXP, EstA, BcAT, CBL, and ArAT activities were measured in cell lysates prepared from cells grown in those media, and the results revealed clear differences in enzymatic activities between the different strains (Figure 2). The results showed that enzyme activity in rich broth medium was not predictive for activity in product environment (see for PepN in Supplementary Figure S3). 

### 3.6. Volatile Composition of Fermented Samples Shows Big Strain Variations

**Figure 3:**
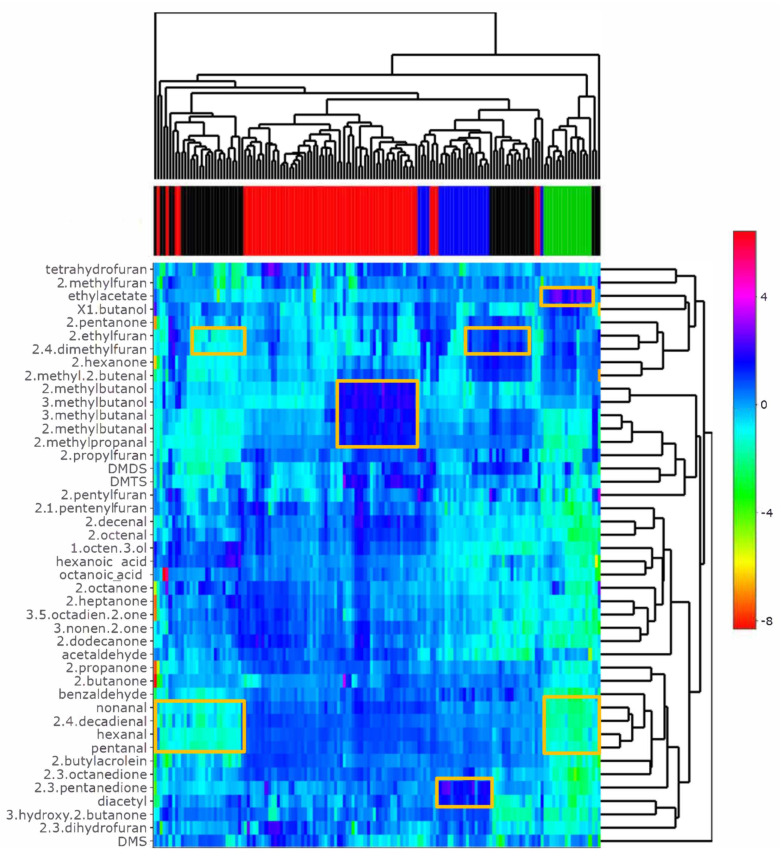
Heatmap of volatile compounds identified after incubation of strains in a mixed emulsion of pea, chickpea, and mung bean protein isolates blended with coconut oil supplemented with 0.5% glucose. Each branch on the dendrogram at the top represents one strain and the top colours in the heatmap indicate the genus of the bacteria, *Lactococcus* (red), *Lactobacillus* (black), *Streptococcus* (blue), and *Leuconostoc* (green). The data were corrected for the blank and transformed to a log10 scale before preparing the heatmap.

Volatile flavour compounds were detected with headspace GC-MS when selected cultures were grown on mixed pea, chickpea, mungbean, and coconut oil emulsion. Figure 3 shows the variation in flavour compound formation by the various LAB species tested. Production of various classes of volatile flavour compounds, such as aldehydes, alcohols, and ketones was clearly species and strain dependent. The removal of typical pea/bean(off) flavour compounds, e.g., of hexanal and furans, was also strain dependent. The rectangular boxes in Figure 3 highlight some specific observations, with clearly lowered n-aldehyde off flavours (pentanal, hexanal, nonanal) when applying lactobacilli and leuconostocs, increased buttery-flavoured diacetyl and pentanedione levels when fermentations took place with streptococci, and highly increased ethylacetate in fermentations carried out with the genus *Leuconostoc*. Branched amino acid degradation products (e.g., 2-methylpropanal, 3-methylbutanal and 2- and 3-methylbutanol) were produced in highest concentrations by a group of lactococcal strains, whereas some of the lactobacilli applied could yield decreased ethyl/methylfurans which are an off-flavour in these plant-based substrates. When zooming in at species within the lactobacilli it was apparent that, especially, *Lactiplantibacillus plantarum* and *Lacticaseibacillus casei* strains could be related to lowered aldehyde and furan levels (Supplementary Figure S5).

## 4. Discussion

The fermentation of milk allows for the production of numerous products, specifically cheeses, with a wide variety of flavours and textures. The application of fermentation on plant substrates to create dairy mimics is growing rapidly and a main challenge is to generate flavours in these non-dairy applications, such as yoghurt, sour cream, and cheese mimics [18]. With the use of plant proteins instead of casein, many challenges arise regarding both the formation of desired cheese-like flavours and the removal of undesired plant off-flavour related compounds. Fermentation has been employed to remove off-flavour notes from plant proteins [2,5,6]. The experiments described in this study focused both on options to remove off-flavour compounds and on the formation of compounds important for cheese flavour when lactic acid bacteria were grown on plant-based substrates.

Acidification and decreasing pH are essential to the process of creating fermented foods, to prevent the growth of spoilage and pathogenic microorganisms. In the present work, acidification experiments were carried out using sterile substrates, which was necessary to prevent the outgrowth of potential spore forming bacteria such as *Bacillus cereus* that could be isolated from the pea protein substrate used. Pasteurisation at lower temperatures of plant protein-based substrates, which was initially used, was not sufficient to prevent the outgrowth of undesired bacteria, such as bacilli. This indicates that a rapid decrease in pH during the fermentation process is likely to play a critical role in the preservation of plant-based food products. During our screening, all plant emulsions were supplemented with glucose. This resulted in a fast decrease in pH with many strains, but there were also strains which performed poorly even with glucose present. Without added glucose, a pH decrease did not occur, showing that the protein material was most likely lacking fermentable sugars. The screening on acidification showed that lactococci tend to reach the lowest final pH values, while streptococci, lactobacilli, and leuconostocs resulted in higher final pH values. When also comparing the acidification rates, *Lactococcus* seemed to perform best, but there was no clear trend between *Streptococcus*, *Lactobacillus*, and *Leuconostoc* species in this assay. The fact that lactococci are better at acidifying plant protein is, to some extent, surprising, as many lactobacilli have an evolutionary history on plant material and are, therefore, expected to grow well on the used substrates [19]. One possible explanation for our finding could be that we added glucose, which can be fermented at a high rate by lactococci. Overall, the data show a broad range of biodiversity within this selected collection of strains, enabling the selection of suitable cultures for proper acidification. Moreover, strains which are able to acidify quickly may allow a limited heat treatment to be applied to the plant substrate as they potentially prevent the outgrowth of pathogenic bacteria. Milder heat treatment should result in better retained techno-functionality of the proteins in the final product.

Compared to the other proteins, (slightly) higher acidification rates and lower pH values were reached on soy-based emulsion, whereas on chickpea, on average, the values were lowest. As all samples tested in our work were supplemented with glucose; the differences observed are likely due to protein compositional differences or other inhibitory molecules. The plant protein preparations used ranged from a protein content of 59% for chickpea up to 88% protein for fava bean. It is known that many LAB are typically auxotrophic, requiring certain amino acids for their growth [20,21], and plant substrates may lack specific amino acids which microorganisms require for their growth. 

To select strains capable of producing desirable plant protein flavour metabolites, e.g., peptides, amino acids, and volatile aroma compounds, 151 strains were screened on production of key peptidolytic and amino acid converting enzymes. The PepN, PepXP, EstA, BcAT, CBL, and ArAT activities measured in lysates of cells grown in BPS supplemented with glucose showed major differences for different strains.

**Figure 4:**
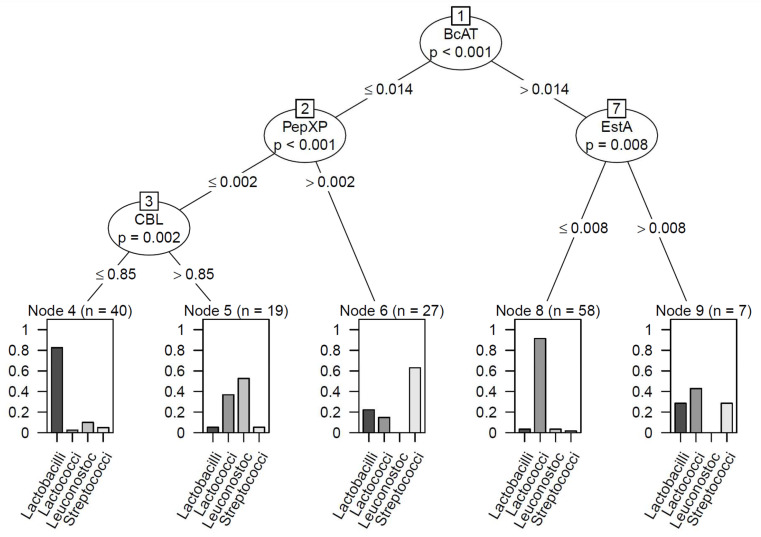
Decision tree to classify bacteria by genus based on enzyme activities measured after growth in BPS supplemented with 0.5% glucose. Example for interpretation: 58 strains fall in Node 8 which is defined as strains where the BcAT activity is higher than 0.014 and the esterase activity is lower than 0.008. Within that node, roughly 90% of all strains are lactococci (error rate with this prediction is lower than 10%).

The clustering per species showed a tendency of higher enzyme activities in lactococci especially when looking at the BcAT activity (Figure 4), whereas relatively low EstA activity made it possible to distinguish lactococci from the other genera (Figure 4). CBL activity could not be correlated with lactococci in general. Lactobacilli expressed in general lower activities of most enzymes quantified when grown in the BPS, while many of the streptococci scored relatively high on pepXP activity (Figure 4). Within the genus *Lactobacillus*, the various strains tested, however, showed quite some diversity, with, for example, (some) *L. helveticus* strains displaying high ArAT and peptidase activities and *L. casei* showing relatively high BcAT and EstA activities (Supplementary Figure S4). The results obtained with lactobacilli, especially, demonstrate differences between dairy and plant substrates with respect to options for choosing suitable cultures for flavour and texture formation. 

Peptidases such as PepN and PepXP are important for releasing amino acids. This is essential for cell growth and biomass formation, but it is also relevant for generating precursors for flavour formation. Abundant research has been carried out to explore the activity of intracellular peptidases of various LAB strains on casein [22,23]. The ability of such peptidases to cleave plant proteins is less understood, but it may, potentially, result in altered and/or insufficient release of amino acids, which consequently may hamper some LAB to grow and colonise plant substrates. The enzymes BcAT, CBL, and ArAT are involved in amino acid converting processes leading to the formation of, often volatile, metabolites that can be important for flavour.

The clear lack of correlation in pepN activity between cells grown in rich broth medium and cells grown in BPS showed that the enzyme activity in rich broth medium may not be predictive for activity in a product environment. Previous research has shown effects of medium and growth rate on the regulation of the peptidase activity, and a regulatory role for specific peptides on the production of the extracellular proteinase PrtP was suggested [24]. Bachmann et al. [12] grew 84 different strains of *Lactococcus* in chemically defined medium and the rich GM-17 medium and found that the enzyme activities measured under certain conditions provides limited to no insight to predict the strains enzyme expression in a different set of conditions. In the current work, the exact protein composition of the media was not determined, and the nature of peptides present was unknown. The BPS was likely less rich in specific peptides. In addition, the emulsions and suspensions used in our experiments were heat treated at 105 °C for 30 min, and the effect of heating at prolonged temperatures may have led to protein aggregation and consequently lower accessibility of the protein. The use of pea protein as the representative pulse protein was an arbitrary choice and further studies exploring the specific enzyme activities that strains express in other plant protein-based media, especially those showing clearly different protein and amino acid compositions, may provide further insight in expression of peptidolytic enzymes. 

The catabolism of proteins into amino acids by proteases and peptidases and, in particular, their further conversion into various aldehydes, (thio)esters, acids, and alcohols is an essential bio-chemical processes for development of flavour and texture during the production and ripening of cheeses [3,25,26]. In particular, the amino acid degrading pathway involving transamination by aminotransferases to a-keto acids and subsequent further conversion of the keto acids to, for example, aldehydes, alcohols, and acids is of importance for the formation of flavour. Esterases can convert alcohols and carboxylic acids into key esters and thioesters respectively, contributing to the overall complexity of cheese aroma [27]. 

Proteins found in plants differ greatly from those in milk. In particular, globular storage proteins belonging to the albumins and globulins are found in legumes including peas, chickpeas mung beans, fava beans, and soybeans, whereas milk contains caseins with much less structure and more flexibility [28,29]. In particular, the presence of the sulphur containing amino acids, cysteine and methionine, of which the latter may be converted to key aromatic compounds such as DMDS and DMTS in cheeses, differs in plant proteins compared to dairy protein. Plant proteins, generally, are deficient in the sulphur-containing amino acids compared to milk and dairy-derived proteins and this is expected to impact the generation of these important metabolites and consequently flavour formation.

The levels of DMDS and DMTS in ferments were relatively high when lactococcal strains were applied. Furthermore, application of some of the *Lactobacillus* species yielded increased DMDS and DMTS levels, most notably *L. helveticus* and *Lacticaseibacillus casei* species. In those cases where DMDS and DMTS were high, the levels of DMS were relatively low. The flavour properties of these volatile sulphur compounds are described as strongly egg, onion, and cabbage-like, but also cheesy and creamy/cooked. The formation in dairy cheeses starts by cleaving methanethiol from methionine by specific lysases, e.g., CBL, or by a methionine transamination process, catalysed by, for example, BcAT, followed by decarboxylation. The degradation of branched-chain amino acids, such as leucine, isoleucine, and valine, also involves action of BcAT followed by decarboxylation of the α-keto acids produced [4]. The resulting branched amino acid degradation products (e.g., 2-methylpropanal, 2- and 3-methylbutanal and corresponding branched-chain alcohols) have typical malty, chocolate-like flavour and their formation in pea emulsion was especially high by various lactococcal strains. The relatively high BcAT activities expressed by most of the lactococci correlate with the level of formation of methionine and branched amino acid degradation products observed.

**Figure 5:**
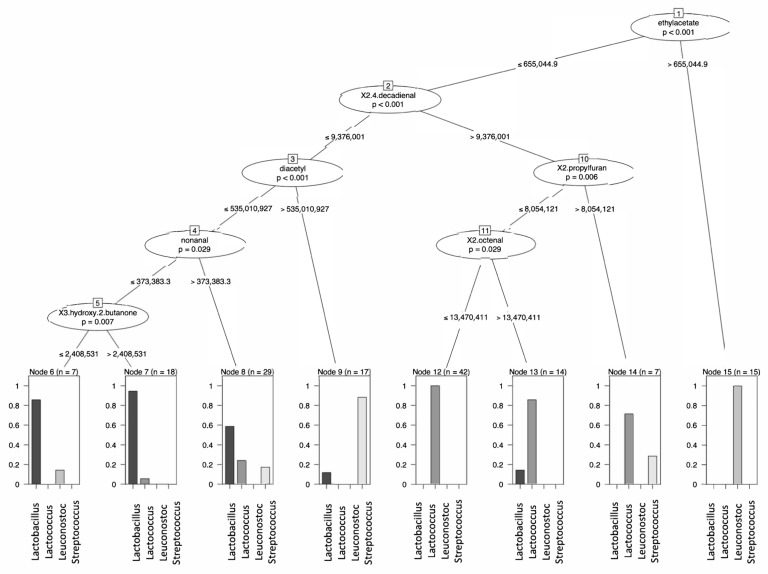
Decision tree to predict the different genera used to ferment a mixture of plant-based proteins based on the production and consumption of volatile compounds.

A decision tree on the GC-MS data to distinguish genera based on volatile compounds showed that the ester ethylacetate was high, and its value made it possible to distinguish the vast majority of leuconostocs from the other species (Figure 5, node 15). Leuconostocs generally possess the capacity to produce ethanol and other alcohols. The formation of ethanol most likely stimulated the formation of ethylacetate. 

The ferments produced by streptococci typically yielded increased levels of the butter-flavoured compounds diacetyl and 2,3-pentanedione. In dairy products, diacetyl is mainly produced by degradation of citrate by *L. lactis* subsp. *lactis* biovar *diacetylactis* and *Leuconostoc* spp. [30]. In the current pea emulsions, the formation of diacetyl and 2,3-pentanedione may have occurred via pathways involving conversion or biosynthesis of, for example, valine, isoleucine, and threonine [31,32]. The formation of the di-ketone compounds has been reported to be important in yogurts where *Streptococcus thermophilus* is involved [27]. Many lactococci are known to possess reductases that catalyse the breakdown of diacetyl. Hugenholtz (1993) noted that a large difference in the diacetyl/acetoin reductase activities exist within the *L. lactis* species [33]. 

Off notes associated with beany and grassy aromas are common in plant proteins and originate from aldehydes such as pentanal, hexanal, and nonanal. These aldehydes are produced from the auto-oxidation and/or enzymatic oxidation of fatty acids, more specifically linolenic and linoleic acids commonly found in plant substrates [34]. Moreover, the formation of unsaturated aldehydes, such as 2,4-decadienal and 2-octenal, are related to lipid/fatty acid conversion processes. Lactococci generally did not lower these compounds, whereas lactobacilli could be classified by lowered levels of these compounds (Figure 5, node 2). Schindler et al. [2], fermented pea protein extracts with *L. plantarum* and *P. pentosaceus* and found that both LAB were effective in decreasing the off notes. Youssef et al. observed similar reduction of green/leguminous attributes when fermenting pea with LAB but also with yeasts [35]. These findings are consistent with our results where Lactobacilli, especially *Lactiplantibacillus plantarum* and *Lacticaseibacillus casei* strains, were effective in decreasing aldehydes such as hexanal and pentanal. Some leuconostocs also decreased levels of -n-aldehydes. 

Besides certain aldehydes, alkylfurans can be found in foods, mainly as a result of thermal processes and/or lipid degradation processes. These compounds may also contribute to plant-based off-flavours. With *Lactiplantibacillus plantarum* and *Lacticaseibacillus casei* strains ethyl and dimethylfuran levels were lowered, whereas, when applying other lactobacilli, e.g., *L. helveticus*, levels remained relatively high. Here, careful selection of suitable cultures may also enable tailoring of flavour formation. 

## 5. Conclusions

This work illustrates how a methodical approach of screening LAB for their acidifying capacity and suitability to produce target aroma compounds and consume off-notes may be utilised for selecting those strains to develop plant-based products with desired properties. As *Lactococcus* strains are ubiquitous and crucial to develop characteristic cheese aromas in Gouda-type as well as other cheeses, it is not surprising that here, multiple *Lactococcus* strains also displayed great potential to form flavours in the plant-based emulsions tested. Other species, e.g., lactobacilli and streptococci, showed interesting capacity to affect flavour formation and off-flavour removal. Further research on enzymes related to lipid catabolism derived flavours would be highly relevant, since these metabolites also play a critical role in the overall aroma profile of cheeses. In addition, the nutritional value can be improved with fermentation through, for example, the production of vitamins and proteolysis, or the removal of antinutritional factors [18]. 

Anticipating an increasing consumer shift towards more plant-based foods and further understanding of fermentative conversions of plant-based substrates will be highly valuable to speed up research and development in this fast-moving market. 

## Data Availability

The raw data presented in the figures of this study are available on request from the corresponding authors.

## Supplementary Materials

The following supporting information can be downloaded at: https://www.mdpi.com/article/10.3390/foods11071005/s1, Figure S1: Raw fluorescence data obtained from high throughput measurements (top panel). Replicate calibration curves to convert fluorescence data to pH values (middle panes). Acidification curves after conversion of raw data; Figure S2: Normalized fluorescence of acidification curves with 11 strains. Strains were incubated in a pea emulsion with and without 0.5% *w*/*v* glucose addition. Without glucose little acidification is seen while with glucose all strains acidify well; Figure S3: PepN activity measured in cell free extract from strains grown in rich broth (left panel) or pea protein suspension (right panel). Each bar represents one strain. In both panels strains are sorted from low to high based on the pepN signal as measured for pea. The data shows poor substrate to substrate predictive value of the pepN measurements; Figure S4: Enzyme activities measured in Lactobacilli after growth in a pea protein emulsion. The side colors in the heatmap indicate the species of the lactobacilli (plantarum black, casei blue, brevis green, pentosus grey, crispatus cyan, acidophilus red, helveticus yellow, delbruecki pink (left panel). The measurements were performed in a pea emulsion.The color legend on the left indicates the scaled (per enzyme) activity from high to low; Figure S5: Heatmap of volatile compounds identified after incubation of lactobacillus strains in a mixed emulsion of pea, chickpea, mungbean, protein isolates blended coconut oil. The top colors in the heatmap indicate the species of the lactobacilli: plantarum (black), casei (blue), helveticus (yellow), acidophlus (red), delbrucki (pink), crispatus (cyan), brevis (green), pentosus (grey). The orange rectangle indicates lower aldehyde and furan levels are found mainly if fermentations too place with L. plantarum and L. casei. The data was corrected for the blank and log10 transformed. The color legend on the left indicates the scaled peak area from high to low.
