# Supplementary material for: Metabolic Conversions by Lactic Acid Bacteria during Plant Protein Fermentations
Source: Foods. 2022 Mar 29;11(7):1005. doi: 10.3390/foods11071005 (PMC8997616; doi:10.3390/foods11071005)
Supplement: Supplementary file 1 [file foods-11-01005-s001.zip › foods-1587051-supplementary.pdf]

Supplementary Material

Metabolic Conversions by Lactic Acid Bacteria during Plant Protein Fermentations

Wim Engels[1]

Jamie Siu[1,2]

Saskia van Schalkwijk[1]

Wilma Wesselink[1]

Simon Jacobs[1]

Herwig Bachmann[1,3]

[1] NIZO, Kernhemseweg 2, 6718 ZB, Ede, The Netherlands

[2] Daiya Foods Inc., 3100 Production Way Burnaby, V5A 4R4, BC, Canada

[3] Vrije Universiteit, Systems Biology Lab, De Boelelaan 1108, 1081 HZ Amsterdam, The Netherlands

Contact: Herwig.Bachmann@nizo.com, Wim.Engels@nizo.com

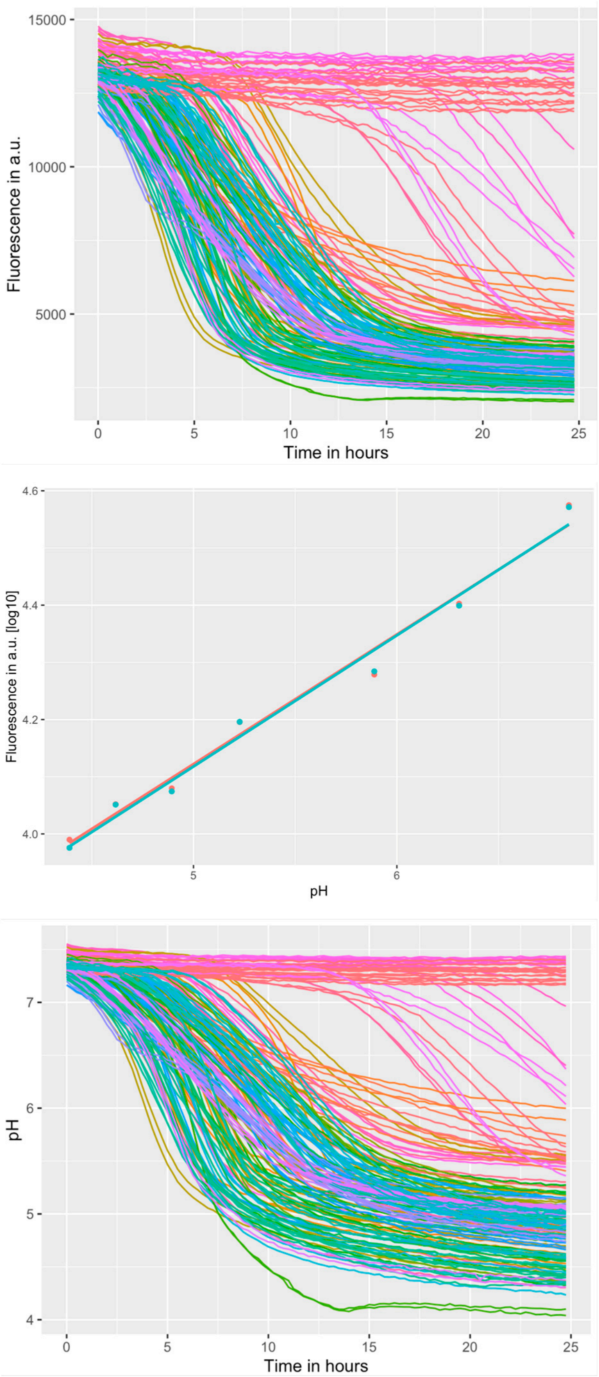

Figure S1: Raw fluorescence data obtained from high throughput measurements (top panel). Replicate calibration curves to convert fluorescence data to pH values (middle panes). Acidification curves after conversion of raw data.

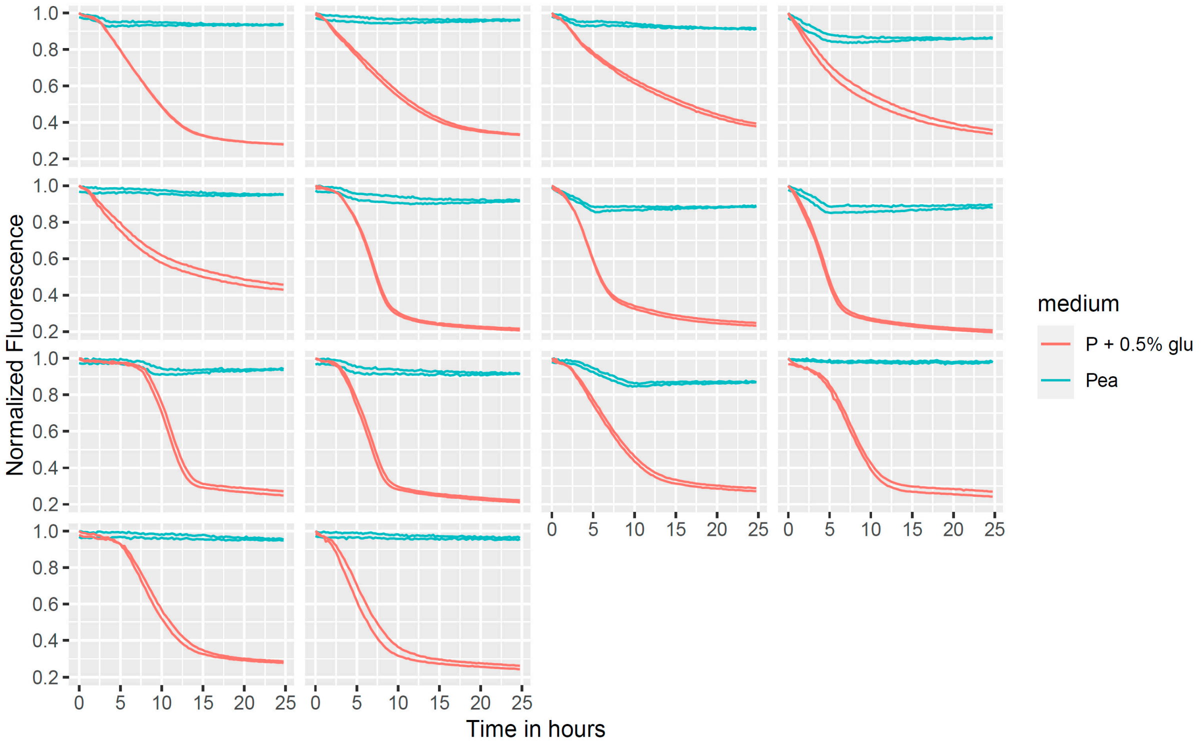

Figure S2: Normalized fluorescence of acidification curves with 11 strains. Strains were incubated in a pea emulsion with and without 0.5% w/v glucose addition. Without glucose little acidification is seen while with glucose all strains acidify well.

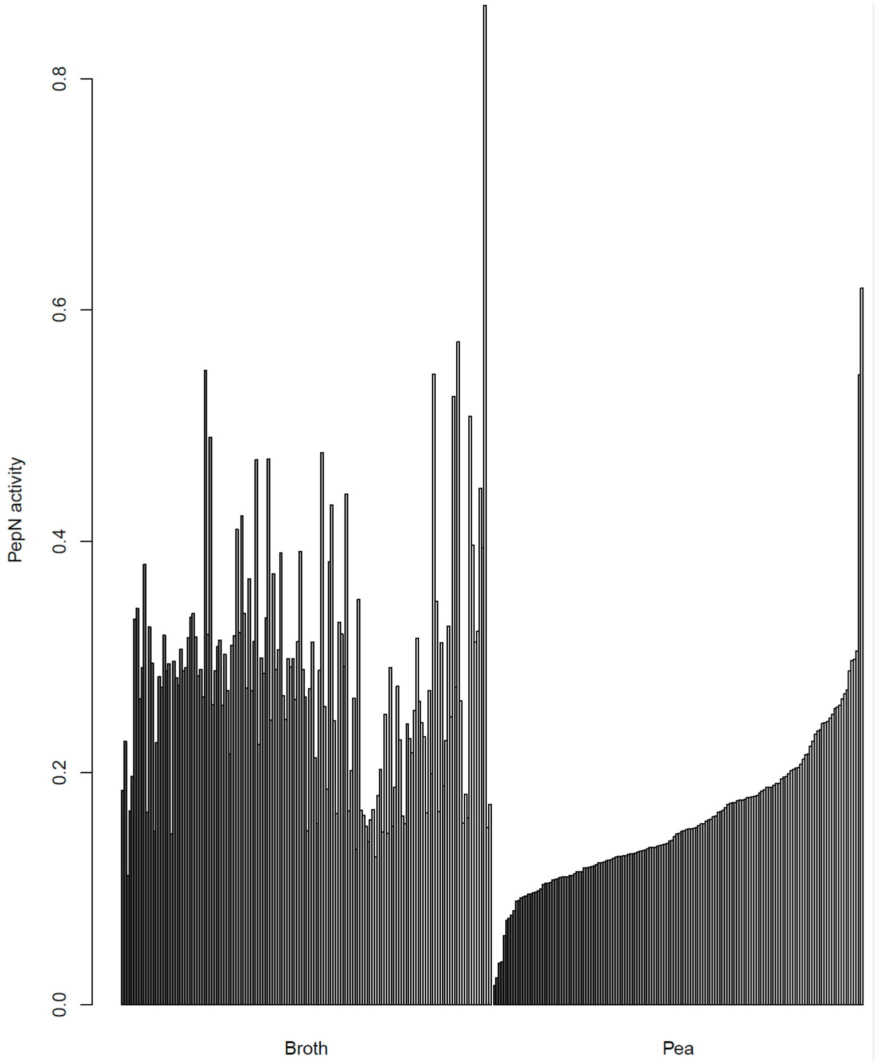

Figure S3: PepN activity measured in cell free extract from strains grown in rich broth (left panel) or pea protein suspension (right panel). Each bar represents one strain. In both panels strains are sorted from low to high based on the pepN signal as measured for pea. The data shows poor substrate to substrate predictive value of the pepN measurements.

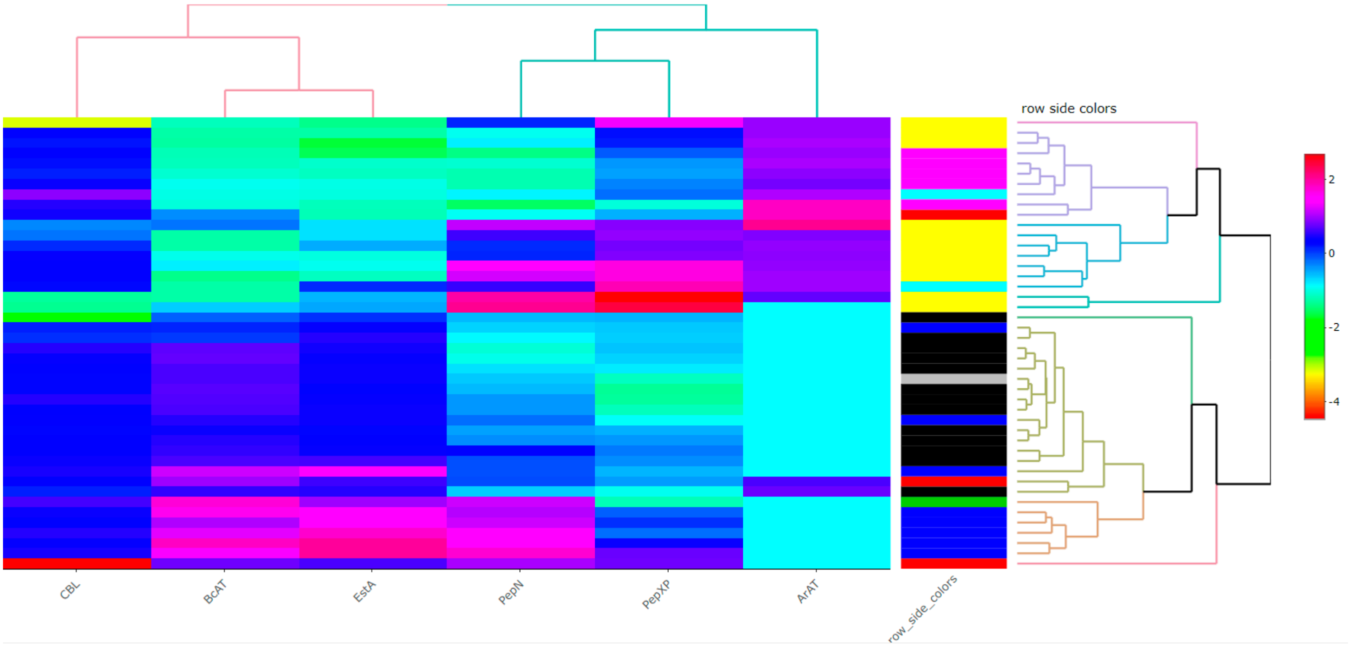

Figure S4: Enzyme activities measured in Lactobacilli after growth in a pea protein emulsion. The side colors in the heatmap indicate the species of the lactobacilli (plantarum black, casei blue, brevis green, pentosus grey, crispatus cyan, acidophilus red, helveticus yellow, delbruecki pink (left panel). The measurements were performed in a pea emulsion.The color legend on the left indicates the scaled (per enzyme) activity from high to low.

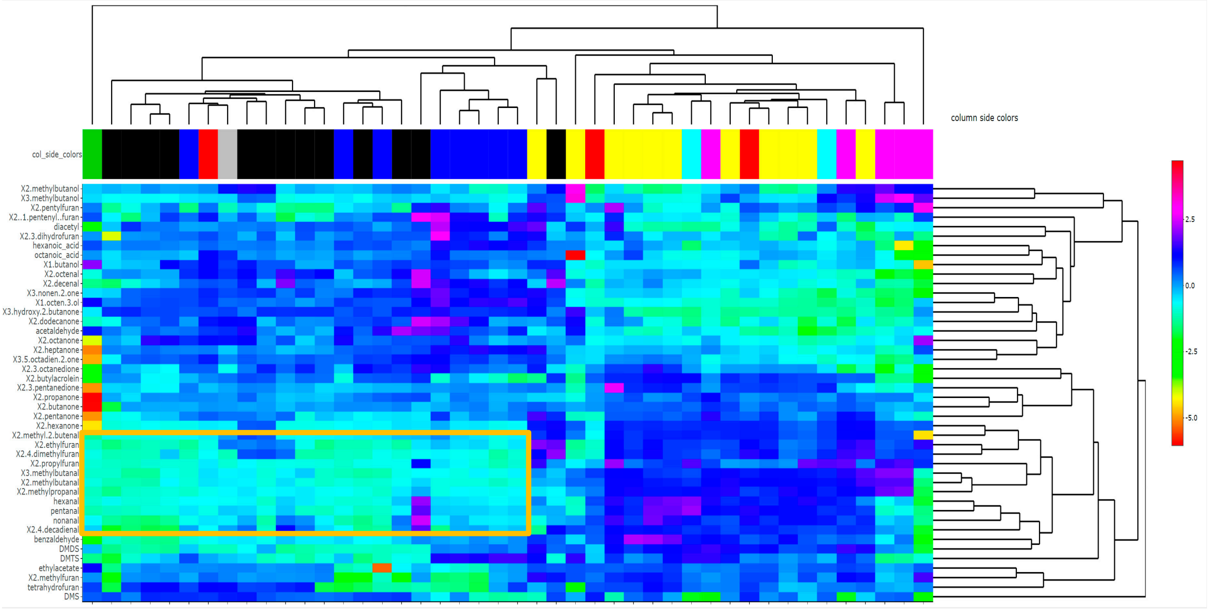

Figure S5: Heatmap of volatile compounds identified after incubation of lactobacillus strains in a mixed emulsion of pea, chickpea, mungbean, protein isolates blended coconut oil. The top colors in the heatmap indicate the species of the lactobacilli: plantarum (black), casei (blue), helveticus (yellow) , acidophlus (red), delbrucki (pink), crispatus (cyan), brevis (green), pentosus (grey). The orange rectangle indicates lower aldehyde and furan levels are found mainly if fermentations too place with L. plantarum and L. casei. The data was corrected for the blank and log10 transformed. The color legend on the left indicates the scaled peak area from high to low.
